# BDNF and TNF-α, OCT and VF Parameters in Pituitary Macroadenoma Patients: A 12-Month Prospective Study

**DOI:** 10.3390/ijms27062609

**Published:** 2026-03-12

**Authors:** Monika Sarnat-Kucharczyk, Beata Kos-Kudła, Małgorzata A. Janik, Paweł Janik, Katarzyna Komosińska-Vassev, Aleksandra Górecka, Ewa Mrukwa-Kominek

**Affiliations:** 1 Department of Ophthalmology, Faculty of Medical Sciences in Katowice, Medical University of Silesia, 40-752 Katowice, Poland; ewa.mrukwa@sum.edu.pl; 2 Kornel Gibinski University Clinical Centre, Medical University of Silesia, 40-514 Katowice, Poland; 3 Department of Endocrinology and Neuroendocrine Tumors, Faculty of Medical Sciences in Zabrze, Medical University of Silesia, 40-514 Katowice, Poland; bkoskudla@sum.edu.pl; 4 Institute of Biomedical Engineering, Faculty of Science and Technology, University of Silesia in Katowice, 41-205 Sosnowiec, Poland; malgorzata.janik@us.edu.pl (M.A.J.); pawel.janik@us.edu.pl (P.J.); 5 Department of Clinical Chemistry and Laboratory Diagnostics, Faculty of Pharmaceutical Sciences in Sosnowiec, Medical University of Silesia in Katowice, 41-200 Sosnowiec, Poland; kvassev@sum.edu.pl (K.K.-V.);

**Keywords:** brain-derived neurotrophic factor (BDNF), tumor necrosis factor alpha (TNF-α), pituitary macroadenomas, optical coherence tomography, visual field

## Abstract

Pituitary macroadenomas often cause visual pathway impairment due to optic chiasm compression. The association between systemic neurotrophic factors and visual recovery remains insufficiently explored. This prospective observational cohort study included 53 patients (106 eyes); 36 patients (72 eyes) completed a 12-month follow-up. Patients were assigned to a treatment group (surgical and/or pharmacological; *n* = 23) or an observation group (*n* = 13). Serum brain-derived neurotrophic factor (BDNF) and tumor necrosis factor-α (TNF-α) were measured at baseline and 12 months. Structural parameters (retinal nerve fiber layer [RNFL], ganglion cell–inner plexiform layer [GCIPL]) and visual field indices (mean sensitivity [MS], mean deviation [MD], square root of loss variance [sLV]) were assessed using optical coherence tomography and automated perimetry. Serum BDNF levels differed significantly between groups at baseline (*p* = 0.0022) and at 12 months (*p* < 0.0001), while TNF-α levels showed no significant changes. The treatment group demonstrated significant improvement in visual field parameters and modest RNFL thickening in the right eye (*p* = 0.0087). Baseline BDNF levels correlated inversely with OCT and visual field measures, particularly in non-functioning adenomas (R = −0.70 to −0.80, *p* < 0.01). Baseline BDNF predicted treatment qualification (AUC = 0.815). Pituitary macroadenomas are associated with visual dysfunction and systemic neurotrophic alterations. Elevated BDNF may reflect a compensatory neuroprotective response, supporting combined molecular and ophthalmic monitoring.

## 1. Introduction

Pituitary gland tumors account for approximately 10–15% of all intracranial neoplasms. The majority are pituitary adenomas (up to 80–90%) [1]. These neoplasms are frequently diagnosed due to clinical manifestations related to hormonal hypersecretion or hyposecretion, or as a result of mass effect on adjacent structures, most notably the optic chiasm. However, some pituitary tumors are discovered incidentally during magnetic resonance imaging (MRI) or computed tomography (CT) performed for unrelated reasons [2].

Brain-derived neurotrophic factor (BDNF) is a member of the neurotrophin family—growth factors essential for neuronal survival, differentiation, and maintenance. It is the most extensively distributed neurotrophin in the central nervous system, with particularly high expression levels in the prefrontal cortex and hippocampus [3].

BDNF is critically implicated in the regulation of the hypothalamic–pituitary–adrenal (HPA) axis during adulthood, exerting a pivotal role in the preservation of physiological homeostasis [4].

A study by Yin and Qi has demonstrated that BDNF plays a pivotal role in promoting tumor progression by accelerating the cell cycle, thereby enhancing the growth of pituitary tumor cells both in vitro and in vivo [5].

Previous immunohistochemical studies have demonstrated expression of neurotrophins and their receptors in GH-secreting pituitary adenomas, supporting a potential role of BDNF-related pathways in pituitary tumor biology [6].

An increasing body of evidence suggests that activation of the BDNF–tropomyosin receptor kinase B signaling pathway in tumor cells initiates a cascade of signaling events that promote cell proliferation, survival, and migration [7]. Although this phenomenon has been primarily described in brain tumors and neuroendocrine neoplasms, there is a biological rationale to suggest that similar mechanisms may also occur in pituitary adenoma cells, particularly in macroadenomas exhibiting an aggressive phenotype [8].

Tumor necrosis factor-alpha (TNF-α) has been implicated in the invasion and progression of pituitary neuroendocrine tumors (PitNETs) [9]. Wu et al. conducted a comparative immunohistochemical study and demonstrated that invasive PitNETs exhibited significantly higher levels of TNF-α expression compared to noninvasive tumors. These findings suggest that TNF-α may play a role in promoting tumor invasiveness [10]. Another study confirmed that TNF-α expression is elevated in PitNETs exhibiting bone invasion. In vitro experiments demonstrated that TNF-α promotes osteoclastogenesis in PitNET cells, leading to bone degradation [11].

Although visual field testing is available as an non-invasive method, it relies on patient cooperation and is inherently subjective [12]. In contrast, optical coherence tomography (OCT) provides objective measurements of the structural parameters of the posterior segment [13,14,15].

To our knowledge, no previous study has simultaneously evaluated OCT, visual field (VF), BDNF, and TNF-α in patients with pituitary macroadenomas. The present study aimed to investigate (i) changes in these parameters over 12 months and (ii) the relationship between biochemical markers and structural or functional measures of visual pathway damage.

## 2. Results

### 2.1. Descriptive Statistics

BCVA remained stable throughout the follow-up period (median 1.0 at baseline and at 12 months; IQR 0.95–1.0), indicating a ceiling effect; therefore, it was not included in the correlation analyses.

Descriptive statistics for BDNF and TNF-α in the treatment and observation groups are presented in Table 1.

Detailed age distributions across study groups and within the treatment group by sex and tumor type are provided in Supplementary Tables S1 and S2.

The distribution of patients according to the 60-year age cut-off in the treatment and observation groups is presented in Supplementary Table S3.

### 2.2. Analytical Statistics

#### 2.2.1. BDNF and TNF-α Concentration (Independent Comparisons)

**Figure 1:**
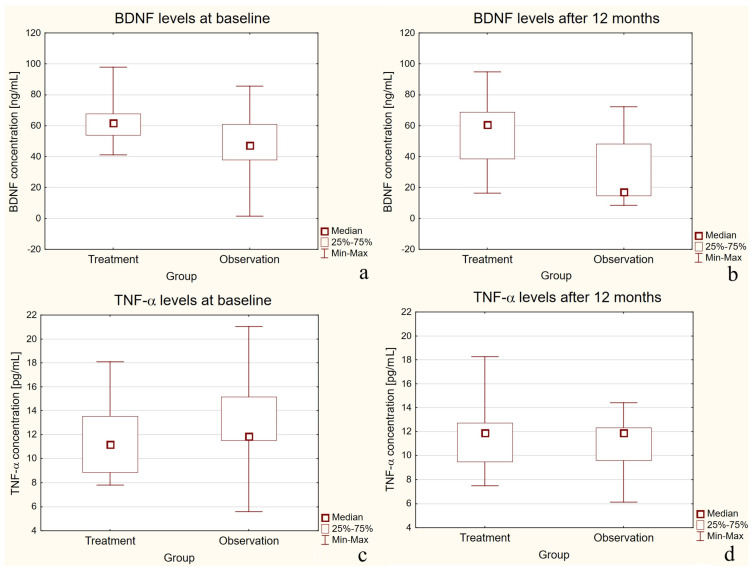
Boxplots of BDNF and TNF-α concentrations in the treatment and observation groups (baseline and 12-month follow-up)—independent comparisons. Figure legend: (**a**) BDNF at baseline; (**b**) BDNF at 12 months; (**c**) TNF-α at baseline; (**d**) TNF-α at 12 months. Box-and-whisker plots show median, interquartile range (25th–75th percentile), and minimum/maximum values for each independent group.

At baseline, serum BDNF levels were significantly higher in the treatment group compared with the observation group (*p* = 0.0022), as presented in Figure 1a. After 12 months, this difference became even more pronounced, with the treatment group maintaining higher BDNF concentrations (*p* < 0.0001) (Figure 1b).

For TNF-α, no statistically significant differences were observed between the two groups at either baseline or after 12 months (*p* > 0.05) (Figure 1c,d).

#### 2.2.2. Changes over Time (Dependent Comparisons)

For BDNF, the difference between baseline and 12-month follow-up measurements was statistically insignificant in the treatment group (*p* = 0.2170) but significant in the observation group (*p* = 0.0024).

In contrast, for TNF-α, no statistically significant difference was observed between baseline and 12-month follow-up in either the treatment group (*p* = 0.9521) or the observation group (*p* = 0.0656), although the latter showed a trend toward statistical significance.

During follow-up, patients in the observation group showed no clinically significant deterioration and none required initiation of treatment.

#### 2.2.3. Ophthalmic Parameters

In both the treatment group and the observation group at both baseline and after 12 months follow-up, there were no statistically significant differences between the right and left eye in terms of mean sensitivity (MS), mean defect (MD), square loss variance (sLV), OCT retinal nerve fiber layer (RNFL) or OCT ganglion cell–inner plexiform layer (GCIPL) values.

Detailed descriptive statistics of the ophthalmic parameters are presented in the Supplementary Materials (Tables S4–S6).

When evaluating changes over time (baseline vs. 12-month follow-up), a statistically significant difference in MS was observed in the treatment group for the right eye (median MS increased from 24.50 [18.50–27.10] at baseline to 26.00 [22.50–27.70] at 12 months; Δ = +1.50) (*p* = 0.0130), while the left eye showed a trend toward significance (median MS increased from 25.40 [15.20–26.70] to 26.10 [17.00–27.30]; Δ = +0.70) (*p* = 0.0742).

In the observation group, a significant difference in MS over time was found only in the right eye (median MS increased from 25.80 [24.50–26.50] at baseline to 26.20 [24.90–27.30] at 12 months; Δ = +0.40) (*p* = 0.0464).

In the treatment group, a statistically significant difference in MD values was observed in the right eye between baseline and after 12-month follow-up (median MD decreased from 3.30 [0.10–7.90] at baseline to 1.60 [−0.10–4.40] at 12 months; Δ = −1.70) (*p* = 0.0111). In the left eye of the same group, there was no statistically significant difference (*p* = 0.1403). In the observation group, no significant differences in MD values were found between baseline and the 12-month follow-up in either eye.

In both groups (treatment and observation), no statistically significant change in sLV values was observed after the 12-month follow-up period. sLV distributions in both groups were highly skewed with visible outliers.

In both groups (treatment and observation), no statistically significant change in OCT GCIPL values was observed after the 12-month follow-up period.

A statistically significant change in OCT RNFL values after 12-month follow-up was observed only in the right eye of the treatment group (median RNFL decreased from 89.00 [85.00–94.00] at baseline to 84.00 [81.00–91.00] at 12 months; Δ = −5.00) (*p* = 0.0049). All other changes were not statistically significant.

#### 2.2.4. Subgroup Analysis

##### Independent Comparisons Between the NFPA and PRLoma Subgroups Within the Treatment Group Showed the Following Findings

**Figure 2:**
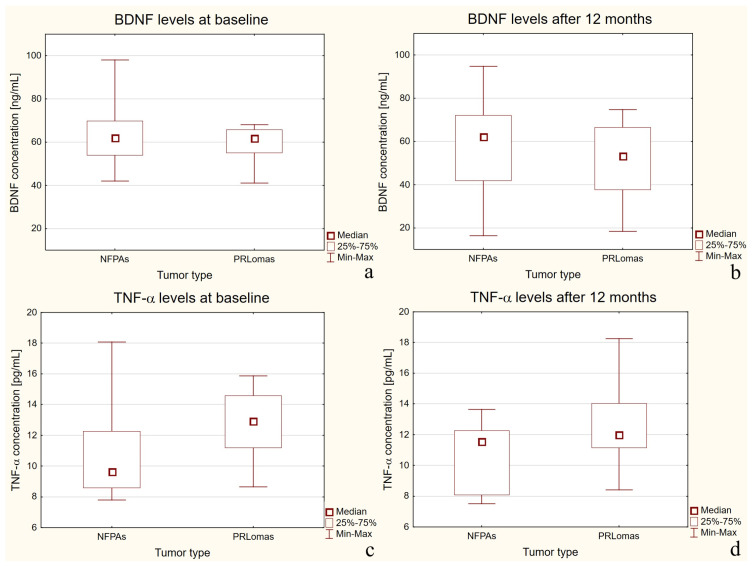
Boxplots of Independent Comparison Between Subgroups: NFPAs vs. PRLoma (Treatment Group Only). Figure legend: (**a**) BDNF concentrations at baseline; (**b**) BDNF concentrations after 12 months; (**c**) TNF-α concentrations at baseline; (**d**) TNF-α concentrations after 12 months.

Within the treatment group, no statistically significant differences in BDNF concentrations were observed between the non-functioning pituitary adenomas (NFPAs) and prolactinomas (PRLoma) subgroups (*p* = 0.6605 at baseline and *p* = 0.4240 after 12 months), as shown in Figure 2a,b.

In contrast, TNF-α concentrations differed significantly between the subgroups at both baseline (*p* = 0.0092) and after 12 months of follow-up (*p* = 0.0314), as presented in Figure 2c,d.

##### Paired (Within-Subgroup) Analyses Assessing Changes over Time Revealed the Following Results

Within the treatment group, in the NFPA subgroup, no statistically significant change in BDNF concentration was observed between baseline and after 12-month follow-up (*p* = 0.2885).

Similarly, in the PRLoma subgroup, BDNF levels did not change significantly over time (*p* = 0.1019). In contrast, a statistically significant increase in BDNF concentration was found in the observation group with NFPA tumors (*p* = 0.0011).

For TNF-α, no statistically significant differences were observed in either for NFPA subgroup (*p* = 0.9637) or PRLoma subgroup (*p* = 0.9133).

In the observation group, the result for TNF-α was also not statistically significant (*p* = 0.0656), although a trend toward statistical significance was observed.

#### 2.2.5. Age Stratification

No statistically significant differences in BDNF levels were observed between baseline and after 12 months of follow-up, either in patients under 60 years of age or those aged 60 years and above in the treatment group. In the subgroup of patients under 60 years with NFPAs, a trend toward statistical significance was noted (*p* = 0.0787). A statistically significant difference was observed in patients under 60 years with PRLoma (*p* = 0.0360). In contrast, BDNF levels remained stable in the older age group, with *p* = 0.8753 for patients with NFPAs and *p* = 0.7989 for those with PRLoma.

There are no statistically significant differences in TNF-α levels between the measurements taken at the beginning and at the end of the observation period, both in the group under 60 years of age and in the group aged 60 and above. For the group under 60 years with NFPAs, *p* = 0.5695; for the group under 60 years with a PRLoma, *p* = 0.3270; for the group aged 60 and above with NFPAs, *p* = 0.4802; and for the group aged 60 and above with a PRLoma, *p* = 0.3329.

#### 2.2.6. Correlations

Significant correlations between retinal parameters and serum concentrations of BDNF and TNF-α were identified exclusively in the treatment group and its subgroups (NFPAs, PRLoma), as presented in Table 2. Notably, several of these associations became more pronounced within the specific treatment subgroups, particularly in NFPA patients, where the correlation coefficients were markedly stronger than in the overall treatment cohort.

In contrast, no significant correlations were detected in the observation group at baseline or at 12-month follow-up.

Most significant associations were observed at baseline, indicating that the relationship between retinal structural measures and neurotrophic or inflammatory biomarkers was already established before treatment initiation. Only one correlation remained statistically significant after 12 months in the treatment group.

The observed correlations demonstrated interocular asymmetry, with OCT and visual field parameters showing significance in the RE, LE, or both.

Significant negative correlations were observed between serum TNF-α levels and OCT parameters reflecting retinal ganglion cell axonal integrity, particularly in patients with NFPAs (R ranging from −0.57 to −0.80, *p* < 0.05). The association was weaker but still present in the overall treatment group, suggesting a relationship between neurodegenerative changes and systemic neurotrophic response.

**Figure 3:**
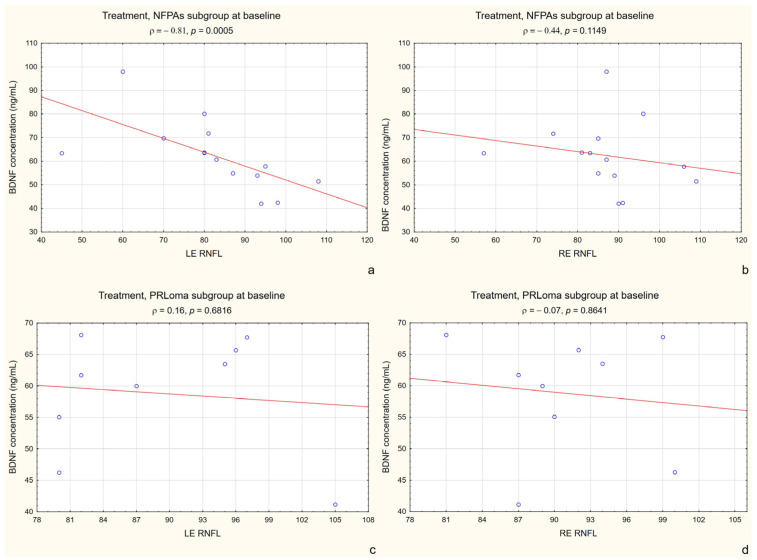
Baseline correlation between BDNF and LE RNFL thickness in the NFPA and PRLoma subgroup at baseline. Figure legend: (**a**) NFPA subgroup (treatment group)—correlation between BDNF and left eye (LE) RNFL thickness at baseline; (**b**) NFPA subgroup—correlation between BDNF and right eye (RE) RNFL thickness at baseline; (**c**) PRLoma subgroup—correlation between BDNF and left eye (LE) RNFL thickness at baseline; (**d**) PRLoma subgroup—correlation between BDNF and right eye (RE) RNFL thickness at baseline. Blue circles represent individual data points, and the red line represents the linear regression line.

At baseline, BDNF levels showed a negative association with LE RNFL thickness in the treatment group (ρ = −0.52, *p* = 0.0117). When the analysis was restricted to the NFPA subgroup, this relationship became markedly stronger (ρ = −0.81, *p* = 0.0005), as illustrated in Figure 3a. No significant correlation was observed between BDNF levels and RE RNFL thickness in the treatment NFPA subgroup (ρ = −0.44, *p* = 0.1149). In the PRLoma subgroup, neither LE RNFL (ρ = 0.16, *p* = 0.6816) nor RE RNFL thickness (ρ = −0.07, *p* = 0.8641) showed a significant association with BDNF concentration at baseline (Figure 3b–d).

**Figure 4:**
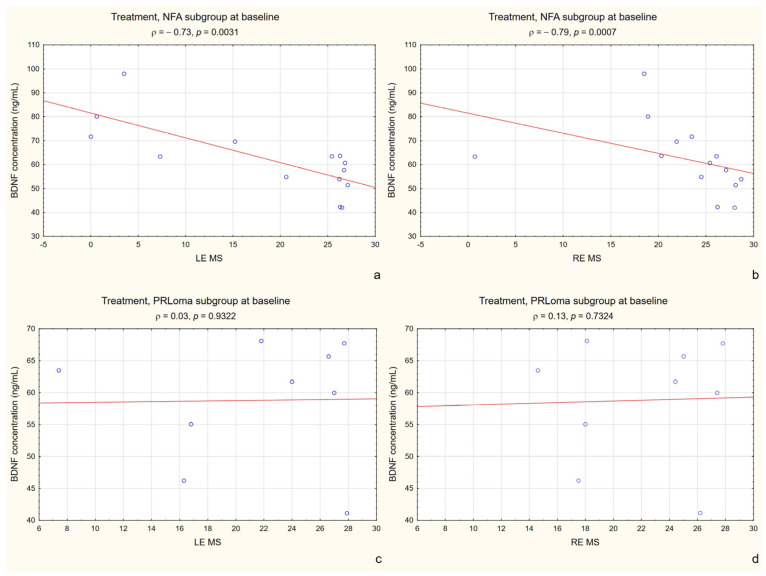
Correlation between BDNF concentration and RE MS at baseline in the NFPA subgroup (treatment group) at baseline. Figure legend: (**a**) NFPA subgroup (treatment group)—correlation between BDNF concentration and left eye (LE) mean sensitivity (MS) at baseline; (**b**) NFPA subgroup (treatment group)—correlation between BDNF concentration and right eye (RE) mean sensitivity (MS) at baseline; (**c**) PRLoma subgroup—correlation between BDNF concentration and left eye (LE) mean sensitivity (MS) at baseline; (**d**) PRLoma subgroup—correlation between BDNF concentration and right eye (RE) mean sensitivity (MS) at baseline. Blue circles represent individual data points, and the red line represents the linear regression line.

A similar enhancement was observed for the correlation between BDNF and RE MS. In the treatment group, baseline BDNF levels showed a moderate negative correlation with RE MS (ρ = −0.43, *p* = 0.039). However, when the analysis was restricted to the NFPA subgroup, this association became substantially stronger (ρ = −0.79, *p* = 0.0007). A significant negative correlation was also observed for LE MS in the NFPA subgroup, whereas no significant associations were detected in the PRLoma subgroup for either eye (Figure 4a–d).

#### 2.2.7. ROC Analysis

ROC analysis was performed to evaluate the ability of serum BDNF levels to discriminate between patients assigned to treatment and observation groups. BDNF values above the identified cutoff were characteristic of the treatment group, whereas lower BDNF levels were characteristic of the observation group.

**Figure 5:**
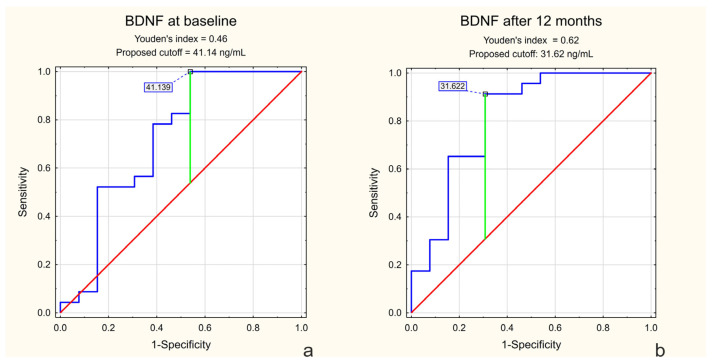
Receiver operating characteristic (ROC) analysis of serum BDNF levels at baseline (**a**) and after 12 months (**b**).

ROC analysis demonstrated that baseline BDNF levels discriminated between patients requiring treatment and those managed conservatively, with an AUC of 0.718 (95% CI: 0.525–0.911; *p* = 0.027), indicating moderate discriminatory ability (Figure 5a). At 12 months, the AUC increased to 0.815 (95% CI: 0.656–0.973; *p* = 0.0001), reflecting stronger between-group separation at follow-up (Figure 5b).

No statistically significant results were observed for TNF-α. Pre-treatment AUC was 0.622 (*p* = 0.2183), and post-treatment AUC was 0.488 (*p* = 0.9065), indicating no diagnostic value in the context of treatment qualification or response.

## 3. Discussion

This study analyzed serum BDNF and TNF-α levels in patients with pituitary macroadenomas during a 12-month follow-up, comparing treatment and observation groups. The results help to understand how treatment may affect neurotrophic and inflammatory responses. By combining biochemical and ophthalmic data (OCT and VF measures), the study explored possible connections between blood biomarkers and visual function recovery.

Taken together with our previous findings [16], the present results support the concept that visual pathway involvement in pituitary macroadenomas may reflect a broader neurobiological process, encompassing both retinal alterations and systemic biomarker changes.

BDNF levels were consistently higher in treated patients, whereas individuals in the observation group showed lower concentrations over time. This pattern may indicate that even hormonally inactive macroadenomas disrupt neurotrophic signaling despite the absence of treatment, possibly due to mechanical pressure or metabolic interference with hypothalamic–pituitary interactions [4]. Differences in BDNF concentrations between treated and untreated patients may suggest that therapeutic intervention modifies the neurotrophic microenvironment [17].

This aligns with prior studies implicating BDNF in tumor microenvironment regulation and pituitary activity [4,18]. Although BDNF may exhibit oncogenic effects in vitro, in vivo studies suggest its role in immune modulation and tumor regression [19,20]. Our findings support the potential relevance of BDNF as a biomarker of treatment response.

In the treatment group, improvement in MS in both eyes likely reflects effective decompression of the optic chiasm following tumor mass reduction, resulting in improved neural conduction and retinal sensitivity [21]. MS and MD appear more sensitive to short-term functional changes than OCT-based structural parameters, which may remain stable over similar timeframes.

Importantly, treatment modalities differed by tumor subtype: prolactinomas were managed with dopamine agonists, whereas most non-functioning adenomas underwent transsphenoidal surgery. These distinct approaches may differentially affect neurotrophic and inflammatory pathways. Surgical decompression likely promotes visual recovery through relief of chiasmatic compression, while pharmacological therapy may additionally modulate systemic neurotrophic signaling. Accordingly, biomarker findings should be interpreted in the context of these heterogeneous clinical interventions.

In the observation group, a significant change in MS was observed only in the right eye, consistent with reports of asymmetric tumor growth and variable optic chiasm involvement [22]. The inherent variability of visual field testing may further contribute to unilateral fluctuations in functional parameters [23].

Despite functional improvements, no significant changes were detected in RNFL or GCIPL thickness over 12 months. This suggests that functional recovery may precede structural changes or occur despite irreversible axonal damage, with possible compensation via neuroplastic mechanisms [24].

The high variability in sLV observed in both groups likely reflects the asymmetric nature of visual field defects in pituitary adenomas. As sLV captures focal sensitivity changes, its variability may be clinically relevant rather than representing measurement error.

In a treatment subgroup, TNF-α levels differed significantly between PRLoma and NFPA patients, both at baseline and after follow-up, suggesting distinct inflammatory profiles related to prolactin secretion [25,26,27]. Prolactin has been shown to directly stimulate TNF-α expression and glial activation, supporting a link between hormonal activity and systemic inflammation [26,28,29,30,31].

BDNF levels remained largely stable across tumor subtypes during follow-up, suggesting resistance to short-term therapeutic modulation. TNF-α levels also showed minimal change, indicating relatively stable systemic inflammatory activity over one year.

Younger patients demonstrated greater variability in BDNF levels, whereas older individuals showed stable concentrations, consistent with age-related reductions in neurotrophin responsiveness [32,33]. No significant age-related effects were observed for TNF-α.

Baseline correlations between serum BDNF levels and ophthalmic parameters suggest a link between neurotrophic signaling and visual function. The inverse relationship between BDNF and OCT measures may reflect compensatory upregulation in response to axonal loss, particularly in NFPAs [34,35].

TNF-α showed associations with OCT parameters, especially in NFPAs, aligning with its established role in retinal neurodegeneration [30]. This supports TNF-α as a potential marker of structural vulnerability rather than treatment response.

ROC analysis demonstrated moderate discriminatory ability of baseline BDNF levels between patients requiring treatment and those managed conservatively. This finding suggests that elevated BDNF may reflect greater disease burden rather than treatment response, consistent with a compensatory neurotrophic reaction in patients with more advanced visual pathway involvement [5]. TNF-α showed no significant discriminatory value.

### 3.1. Study Limitations

This study has several limitations, including a relatively small sample size with loss to follow-up, which may limit statistical power and generalizability. Subgroup analyses were based on small and uneven cohorts, and systemic factors influencing cytokine levels could not be fully controlled. Differences in treatment indications between prolactinomas and NFPAs may also have contributed to baseline clinical heterogeneity between groups. Additionally, the 12-month follow-up may be insufficient to capture long-term structural changes, and the absence of a healthy control group limits interpretation. Another limitation is that biochemical analyses were restricted to patients who completed the 12-month follow-up, as specified in the study protocol. Although blood samples were obtained at baseline from all enrolled individuals, serum BDNF and TNF-α measurements were performed only in participants with complete longitudinal data, which may limit generalizability of baseline biomarker comparisons. Further studies with larger cohorts and extended follow-up are warranted.

### 3.2. Study Innovations

The study provides preliminary evidence that serum neurotrophic markers may be associated with visual pathway involvement in pituitary macroadenomas, suggesting that mechanisms beyond mechanical compression could contribute to visual dysfunction. However, the relative contribution of neurotrophic factors compared to radiological compression was not assessed and requires further investigation.

### 3.3. Future Research Directions

Future studies should confirm these findings in larger, multicenter cohorts, including healthy control groups, to improve generalizability and interpretation of systemic biomarker changes. Longer follow-up periods are needed to determine whether structural retinal changes emerge beyond 12 months and to clarify the temporal relationship between functional recovery and axonal remodeling.

Investigation of additional neuroinflammatory and neuroprotective biomarkers, along with advanced imaging techniques such as OCT angiography or diffusion MRI, may further elucidate visual pathway vulnerability. Finally, mechanistic studies are required to determine whether elevated BDNF reflects a compensatory neuroprotective response or disease severity, and to explore its potential role as a marker of treatment response. Future studies combining detailed radiological metrics with neurotrophic markers such as BDNF may help clarify their joint predictive value for visual pathway damage.

## 4. Materials and Methods

### 4.1. Description of the Study Group

A total of 53 patients (106 eyes) diagnosed with pituitary macroadenomas were initially enrolled in the study. Thirteen patients (26 eyes) were excluded due to loss to follow-up.

Patients with ACTH (adrenocorticotropic hormone; *n* = 2)- and GH (growth hormone; *n* = 2)-secreting pituitary adenomas were excluded from the analysis due to their different hormonal activity profiles and treatment approaches compared with NFPAs and PRLoma. Blood samples were collected at baseline from all enrolled patients. However, according to the predefined study protocol, serum BDNF and TNF-α concentrations were analyzed only in patients who completed the 12-month follow-up. Therefore, biochemical data are available exclusively for this subgroup, whereas baseline ophthalmic examinations were performed in all participants. Thus, 36 patients (72 eyes) completed the 12-month follow-up.

After the initial radiological, endocrinological, ophthalmic and neurosurgical assessments, the patients were divided into two groups. The first group (treatment group) included individuals treated for PitNETs either pharmacologically or surgically, or both, while the second group (observation group) comprised individuals who were observed and did not require treatment.

All included patients had pituitary macroadenomas (tumor diameter ≥ 10 mm), which constituted an inclusion criterion. Tumor size was not analyzed as an independent variable. Treatment allocation was determined following multidisciplinary evaluation based on the overall clinical and radiological presentation rather than tumor size alone.

The treatment group included 23 patients: 9 with prolactinomas and 14 with non-functioning pituitary adenomas.

Within the treatment group 15 patients underwent transsphenoidal surgery, 6 received pharmacological therapy only, and 2 received combined surgical and pharmacological treatment.

Patients with prolactinomas were treated pharmacologically with dopamine agonists (bromocriptine or cabergoline) according to standard clinical protocols.

The observation group comprised 13 patients with only NFPAs, not requiring treatment.

Patients assigned to the observation group had no clinical or radiological indications for intervention at baseline, including absence of significant visual impairment, optic chiasm compression requiring treatment, or evidence of progressive tumor growth. These patients were managed conservatively with active surveillance in accordance with standard clinical practice.

During the 12-month follow-up, patients in the observation group remained clinically and radiologically stable, with no tumor progression or visual deterioration requiring intervention.

**Figure 6:**
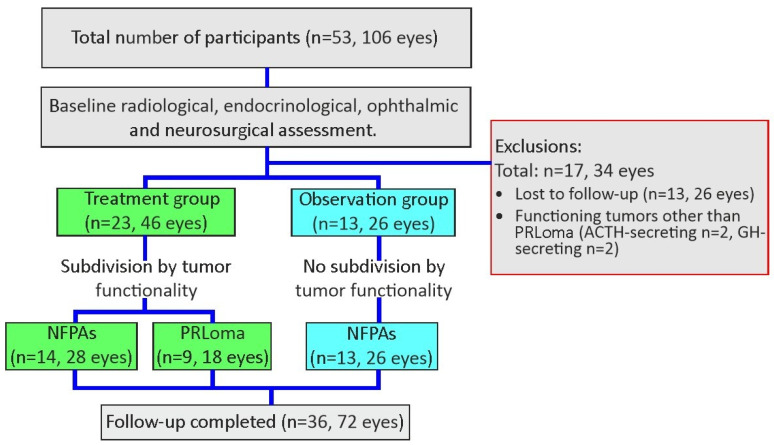
Flow of participants. Figure Legend: Flow diagram of patient selection and grouping. A total of 53 patients (106 eyes) with pituitary macroadenomas were enrolled. After excluding 13 patients (26 eyes) lost to follow-up, 2 patients with ACTH-secreting tumor and 2 patients with GH-secreting tumour,36 patients (72 eyes) were included in the final analysis. Participants were divided into treatment and observation groups and further categorized by tumor functionality (PRLoma and NFPAs). Green boxes indicate the treatment group, while blue boxes indicate the observation group.

The flow of participants is shown in Figure 6.

### 4.2. Laboratory Analysis

#### 4.2.1. Determination of BDNF Levels

Peripheral blood samples were collected from patients diagnosed with pituitary macroadenoma into serum separation tubes. Blood samples were obtained at baseline, before initiation of any pharmacological or surgical treatment; no interim measurements were performed. The samples were allowed to clot at room temperature for 30 min, then centrifuged at 3000 rpm for 5–10 min. The resulting serum was aliquoted and stored at −80 °C until analysis. Serum levels of BDNF were quantified using a commercially available enzyme-linked immunosorbent assay (ELISA) kit (Cloud-Clone Corporation, Katy, TX, USA), based on the sandwich ELISA method. The assay utilizes 96-well microplate pre-coated with capture antibodies specific for human BDNF. Detection was performed using biotin-conjugated anti-BDNF antibodies followed by avidin conjugated to horseradish peroxidase (HRP). After incubation with TMB (3,3′,5,5′-tetramethylbenzidine) substrate, enzyme–substrate reactions resulted in a color change, which was terminated by the addition of stop solution and measured spectrophotometrically at a wavelength of 450 nm. The concentrations of BDNF in samples were calculated using the standard curve comparing the optical density of standards against their concentrations. The sensitivity of the used test was evaluated as 0.055 ng/mL, while the intra-assay precision was lower than 10%.

#### 4.2.2. Determination of TNF-α Levels

Serum levels of TNF-α were assessed using the human TNF-α ELISA kit (BioVendor, Brno, Czech Republic). This test used a sandwich ELISA method, in which the analyzed antigen was immobilized on the plate precoated with ani-TNF-α antibodies. Subsequently, biotin-conjugated detection antibodies and streptavidin-HRP were added to enable the reaction with substrate (TMB) on the microplate. The optical density of the color developed during the enzymatic reaction was measured at a wavelength of 450 nm. The concentration of TNF-α in the samples was calculated using the standard curve derived from recombinant TNF-α standards. Sensitivity of the used test was 2.3 pg/mL, while inter-assay precision was 7.4%.

### 4.3. Ophthalmic Examinations

#### 4.3.1. Best-Corrected Visual Acuity

Best-corrected visual acuity (BCVA) was evaluated at each study visit using standard Snellen charts and initially recorded as decimal values. For statistical analysis, decimal values were converted to logMAR units. BCVA results are presented as medians with interquartile ranges.

BCVA remained stable in both groups throughout the follow-up period (median 0.00 logMAR at baseline and 12 months; detailed data are provided in Supplementary Table S7).

#### 4.3.2. Optical Coherence Tomography (OCT)

OCT was performed using a Zeiss Cirrus 6000 device (Carl Zeiss Meditec AG, Jena, Germany), software version 11.5.2.545332. Peripapillary retinal nerve fiber layer (RNFL) thickness was measured using the Optic Disk Cube 200 × 200 protocol, which acquires a 6 × 6 mm scan centered on the optic nerve head. Ganglion Cell–Inner Plexiform Layer (GCIPL) thickness was assessed with the Macular Cube 512 × 128 protocol (6 × 6 mm), providing automated segmentation of the ganglion cell and inner plexiform layers.

Parameters extracted for analysis included average RNFL thickness and average GCIPL thickness. Scans with poor signal strength (signal strength < 7/10), motion artifacts, segmentation errors, or other quality issues were excluded.

#### 4.3.3. Visual Field

Static visual field testing was performed using the Octopus perimeter (Haag-Streit AG, Köniz, Switzerland). Examinations were conducted with the TOP (Tendency-Oriented Perimetry) strategy, which provides rapid threshold estimation with high test–retest reliability. The analyzed visual field parameters included MS, representing global retinal sensitivity; MD, indicating the average deviation from age-adjusted normative values; and sLV, reflecting local variability and focal defects. All examinations were performed under standard photopic conditions, and tests with low reliability—such as those showing elevated false-positive or false-negative rates—were excluded from the analysis.

### 4.4. Statistical Analysis

The analysis was performed using Statistica 13 (TIBCO Software Inc., Palo Alto, CA, USA, 2017). Statistica (data analysis software system), version 13, https://docs.tibco.com/products/tibco-data-science-statistica-13-5-0 (accessed on 7 March 2024).

#### 4.4.1. Within- and Between-Group Comparisons

Comparisons between baseline and 12-month follow-up within each group were performed using paired statistical tests (paired *t*-test or Wilcoxon signed-rank test, depending on data distribution). Between-group differences (e.g., between age groups or tumor types) were assessed using independent samples tests (Student’s *t*-test or Mann–Whitney U test, depending on data distribution). Normality of distribution was verified using the Shapiro–Wilk test.

#### 4.4.2. Subdivision of the Treatment Group

The treatment group was initially subdivided into PRLoma and NFPAs based on tumor functionality.

#### 4.4.3. Age-Based Stratification

Stratification by age was conducted, using 60 years as the cutoff point.

#### 4.4.4. Correlations Analysis

Correlations between molecular, structural, and functional parameters were evaluated using Spearman correlation coefficients.

#### 4.4.5. Receiver Operating Characteristic (ROC) Analysis

Receiver operating characteristic (ROC) curve analysis was performed to evaluate the diagnostic performance of serum BDNF levels at baseline and after 12 months of treatment. The optimal cutoff value was determined using the Youden index to maximize sensitivity and specificity.

## 5. Conclusions

Pituitary macroadenomas lead to both structural and functional visual pathway impairment, accompanied by alterations in systemic neurotrophic and inflammatory markers. The inverse relationship between OCT parameters and serum BDNF may suggest a compensatory neuroprotective response to optic nerve damage. Combined structural, functional, and biochemical assessment may enhance early detection and monitoring of visual recovery in these patients.

## Figures and Tables

**Table 1:** Descriptive statistics for the treatment and observation groups.

Variable	Timepoint	Group	N	Mean ± SD	Median (IQR)	Minimum	Maximum
BDNF [pg/mL]	0 months	Treatment	23	60.99 ± 12.67	61.73 (53.92–67.75)	41.14	97.99
		Observation	13	46.55 ± 24.16	47.20 (37.84–60.96)	1.56	85.68
	12 months	Treatment	23	55.22 ± 19.09	60.85 (38.47–68.76)	16.43	94.82
		Observation	13	29.29 ± 21.40	17.27 (14.56–48.17)	8.55	72.25
TNF-α [pg/mL]	0 months	Treatment	23	11.42 ± 2.88	11.19 (8.86–13.53)	7.8	18.08
		Observation	13	12.93 ± 3.68	11.86 (11.53–15.14)	5.6	21.04
	12 months	Treatment	23	11.45 ± 2.58	11.92 (9.47–12.69)	7.5	18.25
		Observation	13	11.14 ± 2.24	11.92 (9.58–12.31)	6.14	14.42

Values are reported as mean ± standard deviation (SD) and median (interquartile range, IQR). Concentrations are expressed in pg/mL. BDNF, brain-derived neurotrophic factor; TNF-α, tumor necrosis factor alpha. Timepoints: 0 months = baseline; 12 months = 12-month follow-up.

**Table 2:** All significant correlations between ophthalmic parameters and serum biomarkers in the treatment group and its subgroups (baseline to 12-month follow-up).

Study Group	Ophthalmic Parameter	Eye	Biomarker	ρ (Spearman)	*p* Value
A. Baseline correlations—Treatment group	MS	RE	BDNF	−0.4329	0.0391
	MS	LE	BDNF	−0.5036	0.0143
	sLV	RE	BDNF	0.4495	0.0316
	RNFL	LE	BDNF	−0.5163	0.0117
B. Baseline correlations—NFPA subgroup	MS	RE	BDNF	−0.7934	0.0007
	MS	LE	BDNF	−0.7283	0.0031
	MD	RE	BDNF	0.792	0.0007
	sLV	RE	BDNF	0.8357	0.0002
	GCIPL	LE	BDNF	−0.5425	0.0451
	RNFL	LE	BDNF	−0.5680	0.0341
	RNFL	RE	TNF-α	−0.8057	0.0005
C. Baseline correlations—PRLoma subgroup	GCIPL	LE	TNF-α	0.5174	0.0114
D. Correlations at 12-month follow-up—Treatment group	RNFL	LE	TNF-α	0.5174	0.0114
E. Correlations at 12-month follow-up—PRLoma subgroup	MD	RE	BDNF	−0.6667	0.0499

ρ = Spearman’s rank correlation coefficient. RE, right eye; LE, left eye; MS, mean sensitivity; MD, mean deviation; sLV, square loss variance; RNFL, retinal nerve fiber layer; GCPIL, ganglion cell–inner plexiform layer; BDNF, brain-derived neurotrophic factor; TNF-α, tumor necrosis factor alpha; NFPAs, non-functioning pituitary adenomas; PRLoma, prolactinoma.

## Data Availability

The data presented in this study are available on request from the corresponding author.

## Supplementary Materials

The following supporting information can be downloaded at: https://www.mdpi.com/article/10.3390/ijms27062609/s1.

## Abbreviations

The following abbreviations are used in this manuscript:

ACTH	Adrenocorticotropic hormone
BCVA	Best-corrected visual acuity (BCVA)
BDNF	Brain-derived neurotrophic factor
GCIPL	Ganglion Cell–Inner Plexiform Layer
GH	Growth hormone
HPA	Hypothalamic–pituitary–adrenal
LE	Left eye
MD	Mean deviation
Min-Max	Minimum maximum
MRI	Magnetic resonance imaging
MS	Mean sensitivity
NFPAs	Non-functioning pituitary adenomas
OCT	Optical coherence tomography
PRL	Prolactin
PRLoma	Prolactinoma
RE	Right eye
RNFL	Retinal nerve fiber layer
sLV	Square root of loss variance
TNF-α	Tumor necrosis factor alpha
VF	Visual field
