# Supplementary material for: BDNF and TNF-α, OCT and VF Parameters in Pituitary Macroadenoma Patients: A 12-Month Prospective Study
Source: Int J Mol Sci. 2026 Mar 12;27(6):2609. doi: 10.3390/ijms27062609 (PMC13026532; doi:10.3390/ijms27062609)
Supplement: Supplementary file 1 [file ijms-27-02609-s001.zip › ijms-4131858-supplementary.pdf]

Supplementary Table S1. Age distribution across groups

Group	Gender	N	Mean ± SD	Median (IQR)	Minimum	Maximum
Treatment	Female	11	54.73 ± 13.57	59 (45–68)	30	71
	Male	12	59.83 ± 12.81	59.5 (55–68.5)	31	80
Observation	Female	10	60.20 ± 15.70	64.5 (54–70)	34	83
	Male	2	32.00 ± 9.90	32	25	39

Supplementary Table S2. Treatment group – age by sex and tumor type

Tumor type	Gender	N	Mean ± SD	Median (IQR)	Minimum	Maximum
NFPAs	Female	6	53.83 ± 14.30	58.,5 (45–60)	30	71
	Male	8	60.62 ± 8.12	59.5 (57.5–65)	47	74
PRLomas	Female	5	55.80 ± 14.22	60 (48–68)	35	68
	Male	4	58.25 ± 21.04	61 (42.5–74)	31	80

Supplementary Table S3. Baseline Demographic Characteristics of the Treatment and Observation Groups by Age and Sex

Group	Group by age	Gender	N
Treatment	≥ 60	Female	5
		Male	6
	< 60	Female	6
		Male	6
Observation	≥ 60	Female	6
		Male	-
	< 60	Female	4
		Male	3

Supplementary Table S4. Visual function and retinal structural parameters across treatment and observation groups

Variable	Timepoint	Group	N	Median (IQR)	Minimum	Maximum
MS	0 mths	Treatment	46	24.75 (18.00–26.70)	0.00	28.70
		Observation	26	24.90 (22.80–26.50)	9.20	28.40
	12 mths	Treatment	46	26.05 (20.30–27.50)	0.70	29.90
		Observation	26	25.70 (24.30–27.30)	15.20	30.30
MD	0 mths	Treatment	46	1.95 (0.50–8.30)	-1.50	26.50
		Observation	26	2.20 (0.90–3.80)	-1.10	11.40
	12 mths	Treatment	46	1.55 (0.20–6.20)	-2.70	25.80
		Observation	26	1.50 (0.60–2.20)	-2.90	10.30
LV	0 mths	Treatment	46	4.25 (2.00–5.90)	0.00	73.80
		Observation	26	3.50 (2.60–6.40)	1.30	68.00
	12 mths	Treatment	46	4.05 (2.90–8.10)	0.10	53.70
		Observation	26	4.20 (14.56–48.17)	0.70	57.60
GCIPL	0 mths	Treatment	46	76.50 (70.00–78.00)	38.00	91.00
		Observation	26	77.00 (71.00–81.00)	63.00	89.00
	12 mths	Treatment	46	75.50 (69.00–79.00)	45.00	92.00
		Observation	26	76.00 (72.00–82.00)	67.00	87.00
RNFL	0 mths	Treatment	46	87.00 (81.00–95.00)	45.00	109.00
		Observation	26	83.00 (79.00–93.00)	62.00	113.00

	12 mths	Treatment	46	84.00 (81.00–91.00)	49.00	110.00
		Observation	26	84.00 (78.00–91.00)	71.00	123.00

Supplementary Table S5. Visual function and retinal structural parameters by tumor type in the treatment group

Variable	Timepoint	Group	N	Median (IQR)	Minimum	Maximum
MS	0 mths	PRL	18	24.20 (17.50–27.00)	7.40	27.90
		NFPAs	28	25.40 (18.70–26.60)	0.00	28.70
	12 mths	PRL	18	23.70 (19.30–27.60)	16.30	28.50
		NFPAs	28	26.20 (23.45–27.40)	0.70	29.90
MD	0 mths	PRL	18	2.95 (0.30–8.90)	0.00	19.60
		NFPAs	28	1.35 (0.50–7.55)	-1.50	26.50
	12 mths	PRL	18	2.85 (0.70–7.00)	-0.30	10.10
		NFPAs	28	0.95 (-0.05–3.10)	-2.70	28.80
LV	0 mths	PRL	18	4.55 (2.60–9.90)	0.30	40.60
		NFPAs	28	4.00 (1.85–5.60)	0.00	73.80
	12 mths	PRL	18	5.25 (2.60–10.70)	1.60	47.70
		NFPAs	28	2.50 (1.55–6.75)	0.10	53.70
GCIPL	0 mths	PRL	18	78.00 (71.00–81.00)	60.00	91.00
		NFPAs	28	75.50 (68.50–78.00)	38.00	89.00
	12 mths	PRL	18	78.00 (69.00–81.00)	58.00	92.00
		NFPAs	28	74.50 (68.00–77.50)	45.00	90.00
RNFL	0 mths	PRL	18	89.50 (82.00–96.00)	80.00	105.00
		NFPAs	28	86.00 (80.00–93.50)	45.00	109.00
	12 mths	PRL	18	90.00 (82.00–95.00)	79.00	101.00
		NFPAs	28	83.50 (77.00–88.00)	49.00	110..

Supplementary Table S6. Descriptive statistics of ophthalmic parameters by group, timepoint and eye

Variable	Timepoint	Group	Eye	N	Median (IQR)	Minimum	Maximum
MS	0 mths	Treatment	Right	23	24.50 (18.50–27.10)	0.70	28.70
			Left	23	25.40 (15.20–26.70)	0.00	27.90
		Observation	Right	13	25.80 (24.50–26.50)	9.20	27.90
			Left	13	23.90 (22.70–26.25)	18.40	28.40
	12 mths	Treatment	Right	23	26.00 (22.50–27.70)	0.70	29.90
			Left	23	26.10 (17.00–27.30)	1.20	29.20
		Observation	Right	13	26.20 (24.90–27.30)	15.20	30.00
			Left	13	25.20 (22.95–27.50)	20.00	30.30
MD	0 mths	Treatment	Right	23	3.30(0.10–7.90)	-1.50	25.60
			Left	23	1.60 (0.50–10.20)	0.00	26.50
		Observation	Right	13	1.50 (0.50–2.30)	-1.00	11.40
			Left	13	3.65 (1.20–4.20)	-1.10	9.70
	12 mths	Treatment	Right	23	1.60 (-0.10–4.40)	-2.70	25.50
			Left	23	1.50 (0.30–10.00)	-2.10	25.80
		Observation	Right	13	1.20 (0.00–20)	-1.90	10.30
			Left	13	1.650 (0.70–4.35)	-2.90	7.70
LV	0 mths	Treatment	Right	23	3.80 (1.60–14.50)	0.20	73.80

			Left	23	4.30 (2.00–5.60)	0.00	40.60
		Observation	Right	13	3.40 (2.60–5.60)	1.30	68.00
			Left	13	3.60 (2.55–7.50)	1.80	18.80
	12 mths	Treatment	Right	23	3.90 (1.90–10.70)	0.50	47.70
			Left	23	4.10 (1.70–8.40)	0.10	53.70
		Observation	Right	13	4.10 (3.00–7.20)	1.30	57.60
			Left	13	4.45 (2.75–8.40)	0.70	52.90
GCIPL	0 mths	Treatment	Right	23	77.00 (70.00–78.00)	38.00	88.00
			Left	23	76.00 (69.00-79.00)	55.00	91.00
		Observation	Right	13	77.00 (71.00–81.00)	63.00	89.00
			Left	13	76.50 (73.00–80.50)	68.00	87.00
	12 mths	Treatment	Right	23	76.00 (70.00–79.00)	49.00	88.00
			Left	23	75.00 (69.00–79.00)	45.00	92.00
		Observation	Right	13	78.00 (72.00–81.00)	64.00	87.00
			Left	13	75.50 (73.00–83.00)	66.00	86.00
RNFL	0 mths	Treatment	Right	23	89.00 (85.00–94.00)	57.00	109.00
			Left	23	83.00 (80.00–95.00)	45.00	108.00
		Observation	Right	13	86.00 (81.00–93.00)	62.00	113.00
			Left	13	80.50 (77.00–95.00)	73.00	111.00
	12 mths	Treatment	Right	23	84.00 (81.00–91.00)	50.00	110.00
			Left	23	82.00 (79.00–94.00)	49.00	105.00
		Observation	Right	13	88.00 (83.00-91.00)	71.00	123.00
			Left	13	82.50 (74.50–94.00)	72.00	120.00

Supplementary **Table S7.** Distribution of BCVA (logMAR) by age in treatment and observation groups at baseline and 12-month follow-up

**Group**	**Age**	**Timepoint**	**Median BCVA (logMAR)**	**IQR**
Treatment	<60	0 mths	0.00	0.00–0.02
		12 mths	0.00	0.00–0.00
	≥60	0 mths	0.00	0.00–0.05
		12 mths	0.00	0.00–0.00
Observation	<60	0 mths	0.00	0.00–0.02
		12 mths	0.00	0.00–0.00
	≥60	0 mths	0.00	0.00–0.02
		12 mths	0.00	0.00–0.00
